# Optimizing Intrathecal Opioid Strategies for Cesarean Section: A Comprehensive Narrative Review of Pharmacology, Clinical Outcomes, and Safety

**DOI:** 10.7759/cureus.83109

**Published:** 2025-04-28

**Authors:** Ramona Celia Moisa, Nicoleta Negrut, Mihai Octavian Botea, Teodora Maria Bodog, Cezar Cristian Mihai Moisa, Treesa Clare Thomas, Harrie Toms John

**Affiliations:** 1 Clinic of Anaesthesia and Intensive Care, Pelican Clinic, Medicover Hospital, Oradea, ROU; 2 Doctoral School of Biomedical Sciences/Department of Surgery, Faculty of Medicine and Pharmacy, University of Oradea, Oradea, ROU; 3 Doctoral School of Biomedical Sciences/Department of Psycho-Neuroscience and Recovery, Faculty of Medicine and Pharmacy, University of Oradea, Oradea, ROU; 4 Department of Surgery, Faculty of Medicine and Pharmacy, University of Oradea, Oradea, ROU; 5 Medical School, Faculty of Medicine and Pharmacy, University of Oradea, Oradea, ROU; 6 Critical Care Medicine, Epsom and St. Helier University Hospitals National Health Services (NHS) Trust, London, GBR

**Keywords:** cesarean section, epidural injections, intrathecal opioids, postoperative analgesia, spinal anesthesia

## Abstract

Intrathecal opioids (ITOs), such as morphine, fentanyl, and sufentanil, are widely used as adjuvants in spinal anesthesia for cesarean sections to enhance postoperative analgesia and reduce systemic opioid exposure. Optimizing their selection and dosing is critical to balancing effective analgesia with maternal and fetal safety. This article aims to critically analyze the use of intrathecal opioids in spinal anesthesia for cesarean sections, focusing on their mechanisms of action, clinical benefits, associated risks, and role within Enhanced Recovery After Surgery (ERAS) protocols. A comprehensive narrative review was conducted using PubMed, Scopus, and Google Scholar to identify relevant literature published between January 2010 and March 30, 2025. Morphine provides prolonged postoperative analgesia but is associated with higher rates of pruritus and delayed respiratory depression. Fentanyl and sufentanil offer faster onset but shorter analgesic duration. Combining intrathecal opioids with local anesthetics improves hemodynamic stability and reduces overall opioid requirements. While adverse effects are common, they are dose-dependent and manageable with appropriate monitoring. The tailored selection of intrathecal opioids, guided by pharmacological profiles and patient-specific factors, enhances analgesia and patient-specific safety in cesarean delivery. Refining dosing strategies and integrating multimodal analgesia protocols are essential to minimize adverse effects and optimize maternal and neonatal outcomes.

## Introduction and background

Importance of opioids in spinal anesthesia for cesarean section

Spinal anesthesia remains the preferred choice for cesarean section surgeries because it provides quick results while delivering efficient sensory blocking capabilities. Postoperative pain management, however, remains a challenge, as inadequate analgesia can hinder recovery and affect maternal-infant bonding. The procedure of administering intrathecal opioids (ITOs), which includes morphine, fentanyl, and sufentanil, stands as a common practice for pain relief enhancement alongside decreased systemic effects [1]. Studies show that intrathecal opioids enhance post-cesarean pain management by minimizing supplemental analgesic requirements and reducing the potential for developing chronic pain. Monitoring patient side effects involving itching, nausea, and respiratory depression remains essential. ITO use remains paramount for multimodal analgesia because it supports opioid reduction strategies within Enhanced Recovery After Surgery (ERAS) protocols in spite of potential risks. This review specifically examines how the pharmacological differences between intrathecal opioids influence clinical outcomes and safety in cesarean sections, and evaluates their integration into ERAS protocols.

Mechanism of action of intrathecal opioids

**Figure 1:**
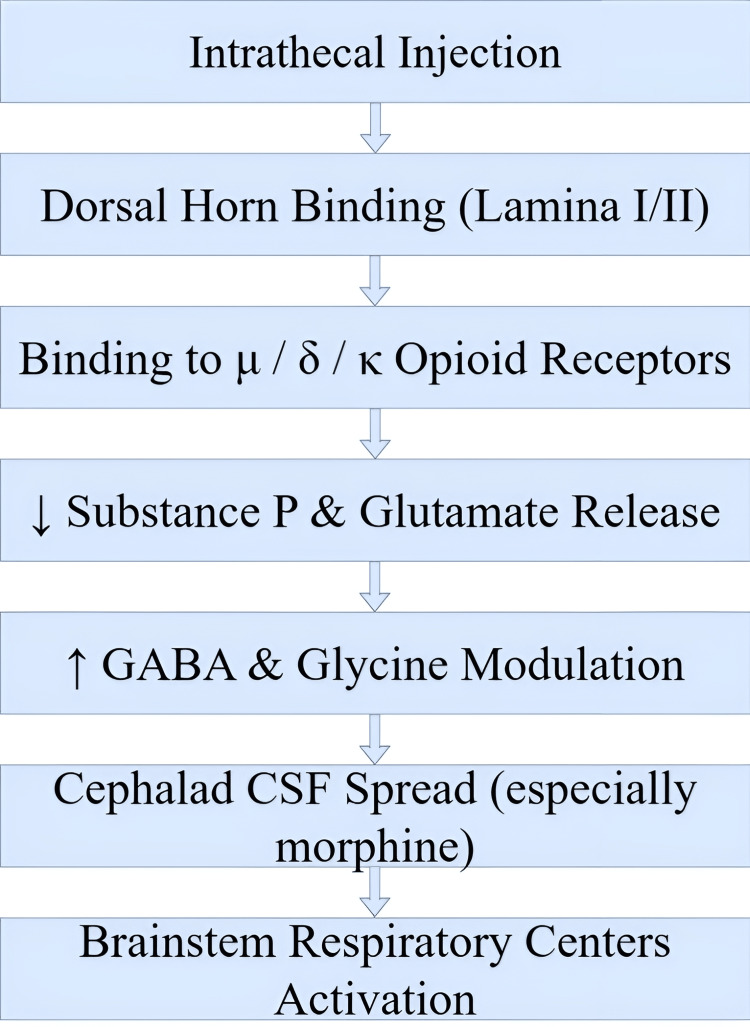
Mechanism of Intrathecal Opioids μ / δ / κ Opioid Receptors: μ (mu), δ (delta), and κ (kappa) subtypes of opioid receptors; Substance P: an 11-amino acid-long neuropeptide expressed by the central nervous system; GABA: gamma-aminobutyric acid; CSF: Cerebrospinal Fluid. The flowchart used here was created by the authors.

When administered intrathecally, intrathecal opioids achieve pain relief through their strong affinity to spinal cord dorsal horn mu-opioid receptors (MORs) [2]. When activated at spinal opioid receptors, these drugs prevent substance P and glutamate from being released, which reduces the transmission of pain signals at low doses while avoiding substantial systemic side effects [2]. The major benefit of intrathecal drug administration lies in its capability to deliver spinal opioid receptor engagement directly, which produces elevated analgesia through low-dose application compared to general opioid use. Through localized delivery, opioids minimize sedative effects alongside respiratory depression side effects [3]. The pharmacological properties of intrathecal opioids differ between morphine and fentanyl and sufentanil because morphine's water-soluble nature leads to slower onset (30-60 minutes) and long-lasting analgesic effects up to 24 hours while fentanyl and sufentanil's fat soluble nature allow faster onset (5-10 minutes) but result in shorter pain control duration [4]. Fentanyl proved more effective during surgery through cesarean spinal anesthesia, but morphine exhibited better postoperative pain relief at the cost of heightened side effect occurrence [5]. These findings emphasize the importance of selecting the appropriate opioid based on the required duration of pain relief and the desired side effect profile. The analgesic mechanism via μ-opioid receptors is detailed in Figure 1. Following injection into the subarachnoid space, opioids bind to the dorsal horn of the spinal cord (lamina I/II), acting primarily on μ, δ, and κ opioid receptors. This interaction suppresses the release of excitatory neurotransmitters such as substance P and glutamate, while enhancing inhibitory modulation via GABA and glycine. In hydrophilic opioids like morphine, cephalad cerebrospinal fluid spread may result in delayed activation of brainstem respiratory centers, contributing to the risk of late-onset respiratory depression.

Rationale for using opioids as adjuvants in spinal anesthesia

**Figure 2:**
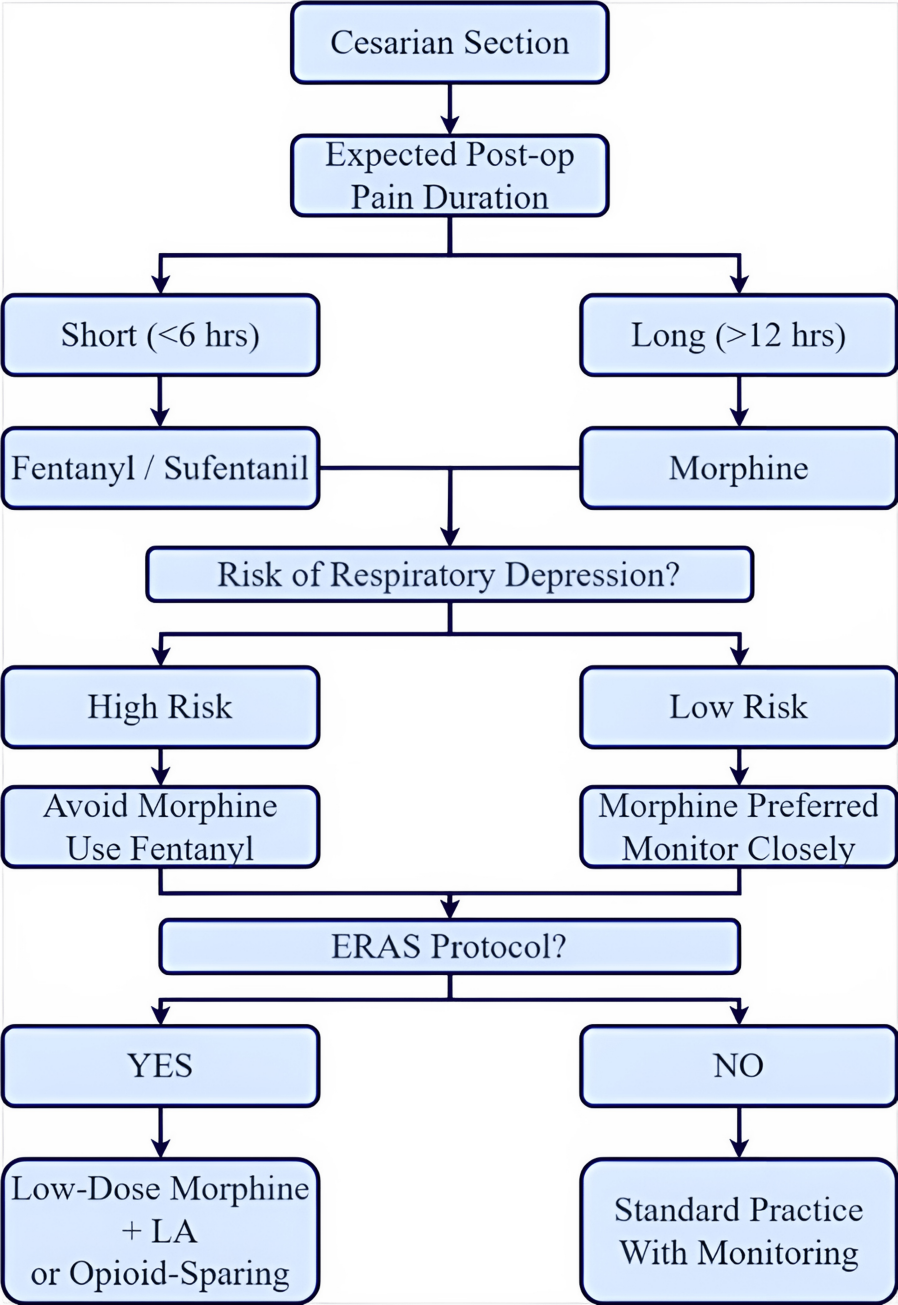
Clinical decision algorithm for intrathecal opioid selection in cesarean section ERAS Protocol: Enhanced Recovery After Surgery Protocol; LA: Local Anaesthetic. The flowchart used here was created by the authors.

Intrathecal opioids are commonly used as adjuvants in spinal anesthesia for cesarean sections to enhance pain relief while addressing the limitations of local anesthetics like bupivacaine, ropivacaine, and lidocaine. While these local anesthetics provide effective surgical anesthesia, their duration is limited. The introduction of intrathecal opioids prolongs sensory blockade without increasing motor block intensity and improves postoperative pain control, leading to faster postoperative mobility [6]. Intrathecal opioid administration reduces systemic opioid usage, thus enabling decreased risks of sedation effects, neonatal exposure, and opioid dependence [7]. Recent research found that patients receiving intrathecal opioids used less systemic opioids postoperatively, reducing opioid-related complications and improving maternal and neonatal outcomes [8]. Additionally, because intrathecal opioids result in lower plasma drug levels compared to systemic opioids, the risk of neonatal respiratory depression is minimized [9]. The combination of local anesthetics and opioids also allows for reduced doses of both, lowering the risk of hypotension and excessive motor blockade. This approach aligns with ERAS guidelines, which promote multimodal, opioid-sparing pain management [10]. Figure 2 illustrates a decision algorithm based on expected pain duration, respiratory depression risk, and ERAS protocol adherence. Fentanyl/sufentanil is preferred for short-duration pain, while morphine is indicated for prolonged analgesia. Selection is further refined by individual risk and institutional opioid-sparing strategies. This framework supports personalised, context-sensitive clinical decisions in obstetric anaesthesia.

The administration of intrathecal opioids provides notable advantages yet requires strategic selection of patients along with accurate medication dosage practices and continuous observation of possible adverse effects. Future research should explore alternative adjuvants and personalized approaches to further improve safety and efficacy in cesarean anesthesia. 

Materials and methods

This narrative review aimed to synthesise current evidence on using ITOs in spinal anesthesia for cesarean section, focusing on pharmacological properties, clinical efficacy, and maternal-fetal safety outcomes. A comprehensive literature search was conducted across PubMed, Scopus, and Google Scholar, covering publications from January 2010 to March 30, 2025. The search strategy included the following terms and their combinations: “intrathecal opioids,” “spinal anesthesia,” “cesarean section,” “morphine,” “fentanyl,” “sufentanil,” “obstetric anesthesia,” and “postoperative analgesia.”

Although this is a narrative review and no formal risk of bias scoring tool (e.g., Critical Appraisal Skills Programme (CASP) or A MeaSurement Tool to Assess systematic Reviews (AMSTAR)) was applied, measures were taken to ensure methodological rigor and reduce selection bias. Two reviewers independently screened all identified titles and abstracts based on the predefined eligibility criteria (Table 1). A full-text evaluation was then conducted to confirm inclusion. Disagreements were resolved through discussion and consensus. Only peer-reviewed articles with clearly reported methodology and clinical relevance to intrathecal opioid use in cesarean section were retained. Studies lacking sufficient methodological detail or clinical data were excluded from the final synthesis.

**Table 1:** Inclusion and exclusion criteria for literature selection

Criteria Type	Criteria
Inclusion Criteria	Peer-reviewed original research, RCTs, systematic reviews, and meta-analyses
	Studies on intrathecal opioid use in obstetric populations
	Articles published in English
Exclusion Criteria	Conference abstracts, editorials, commentaries, and non-peer-reviewed work
	Non-human studies (unless supporting clinical pharmacological insights)

The initial search yielded approximately 70 records. After removing duplicates and assessing titles and abstracts for relevance, 45 articles were selected for detailed analysis. Reference lists of selected papers were also reviewed to identify additional pertinent studies. The final search was completed on March 30, 2025.

## Review

Pharmacokinetics and pharmacodynamics of intrathechal opioids

Proper opioid prescribing necessitates comprehensive knowledge about the pharmacological characteristics and the drug absorption profiles of wide-ranging painkillers. The analgesic substances administered intrathecally interact with G-protein-linked receptors at presynaptic and postsynaptic types present in Laminae I and II regions of the dorsal horn [2,11]. G-protein activation after receptor activation causes mu as well as delta receptors to open potassium channels while kappa receptors close calcium channels, which ultimately decreases intracellular calcium [2,11]. Nociceptive transmission decreases because presynaptic C fiber neurons release less excitatory neurotransmitters (glutamate and substance P) [2]. Opioid receptors at the presynaptic location exist in substantially higher numbers than receptors found postsynaptically [12]. Our postsynaptic receptors in the dorsal horn activate descending brainstem pathways through potassium channel opening when opioid substances bind to these sites [2]. Studies have examined different spinal locations for intrathecal opioid administration [4].

The analgesic effects of fentanyl from the phenylpiperidine opioid family serve as direct local anesthetic actions on C sensory primary afferent nerve fiber endings due to their resemblance to local anesthetics [4]. Studies have shown that administering morphine directly into the spinal canal leads to increased adenosine levels within the lumbosacral cerebrospinal fluid (CSF). When adenosine acts to open potassium channels, it causes neuronal hyperpolarization and a simultaneous reduction of neuronal excitability, which produces analgesic effects [11]. Beyond their interactions with mu-opioid receptors, opioids demonstrate various actions that occur within the spinal cord through distinct mechanisms [11]. The reduction of nociceptive transmission occurs through opioid interference with inhibitory pathways [2,11]. The findings create a fresh understanding of opioid processes within the dorsal horn neural structures.

Intrathecal opioids demonstrate complicated pharmacokinetic properties as they follow a multi-compartmental model that relies on opioid chemical characteristics alongside CSF flow behavior [4]. When considering the pharmacokinetic data for drug distribution in systemic circulation, volume of distribution indicates complete drug mixing and compartment equilibrium between all identifiable areas [4]. The CSF stands as a limited compartment that demonstrates reduced drug distribution [4]. After drug administration into the lumbar CSF space, the development of cephalo-caudal gradients for opioids becomes diminished. After entering the CSF, opioid medications move in a caudal-to-cephalad direction driven by bulk flow. Additional cephalad movement is supported by thoracic pressure fluctuations during respiration and brain volume changes that occur with the cardiac cycle [4]. The movement of CSF along backward and forward pathways enables a total transfer of opioid substance up through the cephalad direction [4]. The posterior radicular artery serves as a pathway for opioid entry into the brainstem by transporting opioid molecules through its uptake process [4]. The analysis of radiolabeled (14C) morphine showed significant radioactivity levels in both spinal cord tissue and respiratory centers after 15-minute and 60-minute post-injection intervals in the lumbar region, respectively [4].

Through studies with animal subjects, Ummenhoffer et al. showed fentanyl's rapid diffusion of the lipophilic opioid into both receptor and non-receptor sites (epidural fat, myelin, and white matter) [4]. The substance achieves wide distribution throughout the spinal cord because of its high octanol: water partition coefficient value at 860 [4]. Only 8% of diffusible fentanyl molecules reach receptor positions in the grey matter because the molecule's high pKa value (8.4) traps most of the drug as it is ionized [4]. The ionized section of the substance remains bound to lipid-rich non-receptor locations [4].

The fentanyl administration leads to fast CSF concentration reduction, followed by increased epidural space concentration and consequent plasma concentration elevation with systemic effects, together with restricted spread to higher segments and localized analgesia [4]. Sufentanil and other lipid-soluble opioids featuring low pKa values and elevated octanol: water coefficients stay active in the spinal cord more extensively which produces extended analgesic outcomes [4].

Morphine stands as one of the most studied hydrophilic opioid opioids administered intrathecally [2,4,9,11]. The hydrophilic nature of morphine reaches 129 to 1737 times the hydrophilic range of fentanyl, even though its octanol: water coefficient is low at 1.4, which slows its diffusion through the epidural space [4,11]. The high-affinity receptors in dorsal horn receptor sites receive more target binding from morphine than non-receptor sites in myelin and white matter of the cord at a lower capacity compared to fentanyl [4]. The limited diffusion volume in the spinal cord maintains high drug levels throughout the cerebrospinal fluid [4]. The clinical observation shows this opioid produces longer-lasting analgesia together with cephalic spread, which can cause delayed respiratory depression [2,4,11]. Morphine infusion into the intrathecal space first establishes a prolonged exposure of the spinal cord, then results in a gradual decrease of CSF drug concentration after 12 hours, which ensures slow drug movement into the epidural area and gradual plasma concentration increases while cephalic distribution can start detecting drugs in cisternal CSF as early as 30 minutes. The drug shows limited circulation in the surrounding cord tissue, and minimal metabolism of water-soluble metabolites occurs in both CSF and spinal cord [4,11]. The administration of radiolabelled (14C) morphine results in a drug retention period of two hours with a remaining percentage of 4.5% after three hours of injection [4]. Drug removal from CSF becomes possible through the glycoprotein carrier transport system present in the choroid plexus [4].

The pharmacokinetic differences outlined in Table 2 have important clinical implications. Morphine’s low lipid solubility and slow CSF clearance result in a delayed onset but prolonged duration of action, making it suitable for extended postoperative analgesia. However, these same properties contribute to its cephalad spread in the CSF, increasing the risk of delayed respiratory depression, particularly relevant in high-risk patients. In contrast, fentanyl and sufentanil exhibit high to very high lipid solubility, enabling rapid onset and fast clearance. Their shorter duration makes them appropriate for intraoperative or short procedures, but less effective for prolonged analgesia. Furthermore, their limited cephalad migration reduces the risk of respiratory depression, improving safety in vulnerable populations. Therefore, the selection of a specific intrathecal opioid should be individualised based on expected pain duration, patient comorbidities, and institutional recovery protocols such as ERAS.

**Table 2:** Pharmacokinetic properties of intrathecal opioids CSF: Cerebrospinal fluid; SC: Subcutaneous; pK_a_: Aid dissociation constant Sources: [2,4,11,12].

Property	Morphine	Fentanyl	Sufentanil
Octanol: Water partition coefficient	~1.4	~860	~1777
pK_a_	7.9	8.4	8.0
CSF clearance time	Slow	Fast	Intermediate
Lipid solubility	Low	High	Very high
Volume of distribution (SC)	Low	High	High

The presented data confirms our current understanding of opioid pharmacokinetics and pharmacodynamics in CSF, but further research must explore the influence of positive pressure ventilation and baricity changes in opioid solutions [2].

Benefits of intrathecal opioids on anesthesia, postoperative analgesia, and post-surgical recovery

The injection of opioids into the intrathecal space combined with local anesthetics remains a prevalent method in obstetric anesthesia because it provides both easy accessibility and operational safety and ease of performance. The combination of surgical and postoperative pain control benefits comes from the administration of long-acting opioids, which can be exemplified by morphine [5]. The combination of opioids with local anesthetics enables practitioners to lower the amount of local anesthetic while preserving pain control efficiency and improving cardiovascular stability. Different types and doses of intrathecal opioids produce variable analgesic outcomes while presenting variable side effect profiles [13].

Morphine remains the most commonly administered neuraxial opioid [6]. According to a survey of Society of Obstetric Anesthesia and Perinatology members performance data showed that most anesthesiologists (87%) choose morphine doses between 50 and 250 μg among which 200 μg is the most commonly (38%) selected dose [14]. A persistent debate exists about whether 100 g or 200 μg morphine produces more effective pain relief or causes more adverse events, according to research that was previously published [15]. The optimal concentration and postoperative analgesic effects for C-section with minimal adverse reactions remain uncertain because available evidence about intrathecal morphine doses has conflicting findings [16]. The research by Sultan Pervez through meta-analysis shows patients receive supplemental analgesia for 4.5 hours longer when using high dose (>100-250 μg) intrathecal morphine over low dose (50-100 μg) [13].

It is important to note that the availability and regulatory approval of intrathecal opioids vary between countries. While fentanyl, sufentanil, and morphine are commonly used globally, diamorphine is frequently utilized in the United Kingdom. Still, it is not available in the United States, Australia, or parts of Eastern Europe. These differences may influence institutional practices and limit the generalizability of study findings across regions.

Research shows that spinal morphine reduces surgical discomfort [16-18], yet other studies claim postoperative effects start only after treatment [19]. According to several clinical trials, spinal administration of 100 mcg to 200 mcg of morphine resulted in intraoperative pain in 18% to 29% of submitted patients [19-22]. Researchers have identified that spinal-administered morphine produces its effects within 30 to 60 minutes, as reported by Fournier et al. [23] and Baraka et al. [24]. The study by Thornton et al. found evidence showing that morphine provided no intraoperative pain relief, which led to 25% of the patients requiring supplemental analgesia during surgery [25]. Studies presented similar results because intraoperative analgesia from morphine administration remains minimal [25,26]. Obstetric anesthesia with fentanyl and local anesthetics produced superior intraoperative anesthesia outcomes but provided inferior postoperative comfort [21,25-27]. Studies in animal models have demonstrated that progesterone has increasing pain-reducing properties on lipophilic spinal opioids throughout pregnancy [10,18]. There are studies that established the equivalence between morphine’s and fentanyl’s potency in generating effective surgical anesthesia [5].

Previous research indicates fentanyl, when mixed with local anesthetics, produces analgesic effects lasting about 12 hours, yet studies show morphine 100 µg generates 18 to 22 hours of analgesic effectiveness [17,26]. A combination of multiple analgesic approaches results in enhanced pain relief and minimizes post-cesarean section opioid medication needs [28]. Table 3 presents a comparison of intrathecal opioid features across pharmacological aspects and clinical effects.

**Table 3:** Comparison of intrathecal opioids: morphine, fentanyl, and sufentanil (pharmacological and clinical characteristics) LA: Local anesthetic; μg: microgram Sources: [5,13,17,25].

Feature	Morphine	Fentanyl	Sufentanil
Solubility	Hydrophilic	Lipophilic	Highly lipophilic
Onset of action	30–60 min	5–10 min	5–10 min
Duration of analgesia	18–24 hours	2–6 hours	4–8 hours
Typical intrathecal dose	50–250 µg (common: 100–200 µg)	10–25 µg	2.5–10 µg
Analgesic potency	Moderate	High	Very high
Cephalad spread	Extensive (increased risk of late respiratory depression)	Limited	Moderate
Respiratory depression risk	High (delayed onset)	Low–moderate (early onset)	Moderate
Pruritus and nausea incidence	High	Moderate	Moderate
Hemodynamic stability	Neutral	Improves with LA synergy	Similar to fentanyl
Intraoperative analgesia	Limited (mostly postoperative)	Effective	Effective
Preferred use	Postoperative pain control	Intraoperative and early recovery	Intraoperative and longer recovery

The effects of intrathecal opioids on the hemodynamic stability of the mother

Intrathecal opioids are commonly used as adjuvants in spinal anesthesia for cesarean sections to enhance analgesia and intraoperative comfort without compromising maternal hemodynamic stability. This effect is achieved through the synergistic interaction between opioids and local anesthetics, leading to effective anesthesia with fewer side effects [29].

Firstly, the addition of intrathecal opioids allows for a reduction in the dose of local anesthetics required to achieve effective anesthesia. This synergy decreases the need for additional systemic analgesics, which might otherwise affect hemodynamic stability during cesarean sections. In their study, Gerbershangen and Baagil concluded that reducing local anesthetic usage is associated with fewer hemodynamic fluctuations [30]. The study by Botea et al. demonstrated that intrathecal fentanyl served as an effective adjuvant by providing fast blockade establishment with reduced procedural time while enhancing spinal anesthesia quality [5]. The short duration of onset enables fentanyl to function as a bupivacaine substitute, thus reducing the occurrence of hypotension when performing surgery [5].

In the study by Coviello et al., which compared the use of sufentanil and dexmedetomidine as adjuvants in spinal anesthesia, no significant difference was found between the groups regarding the incidence of hypotensive events. However, α2-agonists are known for their potential effects on hemodynamic variables, though these changes are dose-dependent [29].

Spinal anesthesia, commonly used during cesarean sections, induces a sympathetic blockade by inhibiting the transmission of nerve impulses through the sympathetic nervous system [31,32]. This blockade leads to vasodilation, resulting in maternal hypotension, which can compromise uterine blood flow and fetal circulation, potentially causing fetal hypoxia and acidosis [5,6]. Another feature of intrathecal opioids as adjuvants is that by lowering the dosage of local anesthetics, they result in a less extensive sympathetic blockade. This moderation helps maintain vascular tone and blood pressure, thereby contributing to hemodynamic stability [31-33]. Lastly, as adjuvants, they help maintain a stable heart rate. By minimizing the dose of local anesthetics, they reduce the incidence of bradycardia. This effect is crucial in maintaining cardiac output and overall hemodynamic balance during the perioperative period [30-35].

Incorporating intrathecal opioids in spinal anesthesia for cesarean sections not only offers effective analgesia but also plays a significant role in preserving maternal hemodynamic stability. This leads to improved patient satisfaction and better surgical outcomes.

The impact of intrathecal opioids on fetal parameters

As the use of intrathecal opioids is common, understanding their impact on fetal parameters is essential for ensuring neonatal safety. One of the investigated parameters was fetal heart rate and contraction monitoring. In the study by Wei et al., maternal heart rate, blood pressure, fetal heart rate, and contraction intensity were monitored [36]. Patients in their study received a mixture of fentanyl and ropivacaine. They concluded that this analgesic approach effectively managed labor pain without adversely affecting fetal heart rate patterns, suggesting a minimal impact on fetal well-being [36]. Another evaluated fetal parameter was the Apgar score. The study by Coviello et al. compared two groups of patients: one receiving sufentanil as an intrathecal adjuvant and the other receiving dexmedetomidine. Ultimately, there was no statistically significant difference in Apgar scores between the groups, leading to the conclusion that these agents do not negatively influence immediate neonatal health [29].

A third parameter considered was respiratory depression and neurobehavioral effects. The ability of opioids to cross the blood-placental barrier raises concerns about potential neonatal respiratory depression. However, this impact varies depending on the specific opioid and its dosage. In the study by Mota-Rojas et al., fentanyl was compared to morphine as an adjuvant. Due to its higher liposolubility, fentanyl exhibited a greater transplacental transfer rate than morphine. However, this potential issue may be mitigated with appropriate dosing [37]. Lastly, regarding fetal stress response and cardiovascular effects, studies have shown that in short procedures, appropriate dosing is likely to proceed without significant side effects [38]. 

While current evidence supports the short-term safety of intrathecal opioids concerning Apgar scores, heart rate, and respiratory parameters, long-term neurodevelopmental and behavioural outcomes remain insufficiently studied. Most clinical trials focus on immediate postnatal parameters, with limited longitudinal follow-up. Given the theoretical risk of neurotoxicity and the known transplacental passage of some opioids, further prospective studies are needed to evaluate potential long-term cognitive, behavioural, and emotional effects on neonates exposed to intrathecal opioids during delivery.

Considering all of the facts, current evidence suggests that when correctly administered, intrathecal opioids as adjuvants do not negatively affect fetal parameters such as Apgar score, heart rate, respiratory function, or neurobehavioral outcomes. It is essential to use the appropriate agent, at the correct dose and timing, to minimize any risks to the fetus. Continuous fetal monitoring during labor and delivery remains a critical component of maternal and fetal care.

Adverse reactions and complications associated with intrathecal opioids

Intrathecal opioids are widely used for effective pain management; however, their usage may cause adverse reactions and complications. Understanding the potential side effects is crucial for improving patient care and ensuring safety. One of the main adverse reactions is respiratory depression. Intrathecal opioids, specifically morphine, can cause respiratory depression due to their migration in the cerebrospinal fluid (CSF), affecting respiratory centers in the brainstem. This necessitates vigilant postoperative monitoring to promptly identify and manage this effect [5,29,39].

Pruritus is another common side effect associated with intrathecal opioid administration. The exact pathophysiology of opioid-induced pruritus remains incompletely understood, but some proposed mechanisms include central opioid receptor activation, serotonergic pathway modulation, and prostaglandin release [40]. In cesarean sections, the occurrence varies from 30-60%, with some populations reporting incidence rates as high as 88% [41,42]. The incidence and severity depend on the opioid used, its dosage, and individual patient susceptibility [43].

Other side effects include nausea and vomiting, which are frequently reported with intrathecal opioid use [41]. Additionally, urinary retention may be caused by intrathecal opioids, sometimes necessitating catheterization [44,45]. Hypotension can also occur as an adverse effect. By reducing sympathetic tone, opioids can contribute to hypotension, even though this is primarily associated with local anesthetics [29,41]. Risk factors that can lead to hypotension include the addition of opioids in spinal anesthesia, properties of the opioid used (some opioids are more lipophilic), high spinal blocks, dehydration, and multiple gestation pregnancies [29]. This risk can be mitigated with close monitoring and appropriate management strategies.

A rare but significant side effect is neurological complications, such as dizziness and, in severe cases, neurotoxicity. These risks highlight the importance of appropriate dosing and vigilant monitoring to prevent severe neurological adverse events [9]. While current evidence supports the short-term safety of intrathecal opioids concerning Apgar scores, heart rate, and respiratory parameters, long-term neurodevelopmental and behavioural outcomes remain insufficiently studied. Most clinical trials focus on immediate postnatal parameters, with limited longitudinal follow-up. Given the theoretical risk of neurotoxicity and the known transplacental passage of some opioids, further prospective studies are needed to evaluate potential long-term cognitive, behavioural, and emotional effects on neonates exposed to intrathecal opioids during delivery.

To facilitate clinical interpretation, Table 4 summarises the most commonly reported adverse effects associated with intrathecal opioids used in cesarean section. The table compares the severity and frequency of key complications, such as respiratory depression, pruritus, and urinary retention, across morphine, fentanyl, and sufentanil. Each opioid’s pharmacokinetic profile influences these differences and should guide anaesthetic decision-making, especially in high-risk patients.

**Table 4:** Adverse effects of intrathecal opioids LA: Local anesthetic; 24h: 24 hours Sources: [5,29,39,41,43].

Adverse Effect	Morphine	Fentanyl	Sufentanil	Notes
Respiratory depression	High (delayed)	Low	Moderate	Monitor for 24h with morphine
Pruritus	High	Moderate	Moderate	30–60% incidence with morphine
Nausea/Vomiting	Moderate–High	Moderate	Moderate	Dose-related
Hypotension	Mild	Minimal	Minimal	Mostly due to synergy with LAs
Urinary retention	Moderate	Low	Low	Especially with morphine

Table 5 presents a comparative summary of common adverse effects associated with intrathecal opioids, highlighting the estimated incidence rates and key pharmacological differences. The data underscore the importance of tailored drug selection to optimize maternal safety while maintaining effective analgesia.

**Table 5:** Summary of adverse effects and estimated incidence of intrathecal opioids Sources: [5,29,39-45].

Adverse Effect	Morphine	Fentanyl	Sufentanil	Estimated Incidence (%)	Notes
Respiratory depression	High (delayed)	Low	Moderate	0.1–7% (morphine); <1% others	Monitor for 24h with morphine
Pruritus	High	Moderate	Moderate	30–88% (morphine); 20–30% others	Higher with hydrophilic opioids
Nausea/Vomiting	Moderate–High	Moderate	Moderate	20–40% (morphine); 15–25% others	Often dose-dependent
Hypotension	Mild	Minimal	Minimal	5–10%	Mostly due to synergy with local anesthetics
Urinary retention	Moderate	Low	Low	5–30%	Especially with morphine

Intrathecal opioids are effective adjuvants in enhancing analgesia for cesarean sections. However, close monitoring and correct patient management are essential, as they may be associated with several potential adverse effects that, in some cases, could lead to a negative prognosis.

## Conclusions

This review synthesizes current knowledge on intrathecal opioid use in obstetric anesthesia, emphasizing pharmacologic distinctions, comparative adverse effects, and implications for early ambulation. While lipophilic opioids like fentanyl and sufentanil may support earlier mobilization due to shorter action and fewer side effects, morphine’s prolonged analgesia is offset by higher rates of pruritus and urinary retention. Limitations include the lack of formal bias assessment, heterogeneity across studies, and limited data on long-term outcomes. Future research should refine multimodal analgesia protocols and improve safety monitoring to optimize recovery within Enhanced Recovery After Surgery (ERAS) pathways.
